# Enhancing District Health Center membership among older adults through intergenerational home-based and AI-generated digital interventions: protocol for a pilot cluster randomized controlled trial

**DOI:** 10.3389/fpubh.2026.1832388

**Published:** 2026-07-24

**Authors:** Aaron Wan Jia He, Ferrina Hoi Yan Cheung, Yuna Shao, Matthew Siu Long Chau, Sophia Siu Chee Chan

**Affiliations:** School of Public Health, Li Ka Shing Faculty of Medicine, University of Hong Kong, Hong Kong, Hong Kong SAR, China

**Keywords:** artificial intelligence, health policy, Hong Kong, older adults, primary health care, public health

## Abstract

**Background:**

Hong Kong is experiencing rapid population aging, with the population of older adults expected to reach 36.0% by 2046. The rise in life expectancy is often accompanied by increased chronic diseases and mental health challenges. The healthcare system remains heavily focused on tertiary care, leading to increased system burden and suboptimal health outcomes. Strengthening engagement with primary care for older adults, particularly through community-based services such as District Health Centers, is essential for improving long-term health outcomes. This approach promotes early detection, better management of chronic conditions, and prevention of unnecessary hospitalizations.

**Objective:**

This pilot study aims to evaluate the feasibility, acceptability, and preliminary effectiveness of a hybrid intergenerational home-based and AI-generated digital intervention for increasing the proportion of District Health Center members among older adults in Hong Kong.

**Methods:**

This is a two-group parallel cluster randomized controlled trial, involving 180 older adults recruited from 6 clusters. Participants will receive home-based DHC intervention by trained students, followed by 1-month of AI-generated digital interventions, including infographics, videos, and messages on the introduction and promotion of DHC, via WhatsApp. The feasibility indicators include recruitment rate, retention rates, dose delivered, dose received, and participant engagement. Acceptability outcomes are workshop and digital intervention satisfaction. The primary outcome is to increase the proportion of District Health Center members. Secondary outcomes include the proportion of participants utilizing DHC, health status, behavioral modifiable risk factors, eHealth literacy, infectious disease prevention behaviors, and feasibility metrics. Data will be collected via surveys at baseline and follow-ups (1, 3, and 6 months).

**Discussion:**

This proposed pilot study aims to use hybrid intergenerational home-based and AI-generated digital intervention to improve primary healthcare engagement among older adults in Hong Kong. By promoting awareness and utilization of primary healthcare services like District Health Centers, the study seeks to promote healthier aging, improve health outcomes, alleviate hospital strain, and provide a framework for addressing the needs of aging populations.

**Clinical trial registration:**

https://clinicaltrials.gov/, NCT07252934.

## Introduction

The global aging population is rising rapidly, with one in six people expected to be aged 60 years or older by 2030 (1). In Hong Kong, this situation is one of the most advanced in the world, with the proportion of older adults projected to reach 36.0% by 2046 (2). Over 70% of this large demographic are affected by chronic diseases, and among the most common are hypertension (57.4%) and diabetes mellitus (19.0%) (2–4). These challenges bring increased health service demand, rising medical costs, manpower shortages, and strain on the healthcare system (5). The total health expenditure has grown to 10.0% of GDP in 2022/2023 and is continuing to grow at an annual rate of 5.6% (4, 6). To address these pressing aging challenges and ensure a sustainable healthcare system, a primary healthcare focused approach should be used.

The Hong Kong population has a health-seeking culture that emphasize heavily on acute, episodic medical care, resulting in only 60% of the population maintaining a regular healthcare service (7, 8). This leads to the high reliance on secondary and tertiary care in the healthcare system, with 65.5% of health resources allocated to these sectors (4, 6). To mitigate the overutilization of acute care services, the Government has been working toward a shift from a treatment-focused to a prevention-oriented care (8). Since 2019, District Health Centers (DHCs), have been set up in all 18 districts in Hong Kong. These centers provide primary healthcare services across four categories, including health promotion, health assessment, chronic disease management, and community rehabilitation services (9, 10). The enrolment these primary care centers are vital to initiate the continuity and coordination of care by a professional, interdisciplinary healthcare team (11, 12). It supports systematic long-term health management through advisory health counseling, early detection, and timely treatment (13, 14), which is particularly well-suited to the needs of the older adult population with prevalent chronic conditions (2–4). However, our previous community study of 7,531 older adults showed a low membership rate of 28.9% for DHC, limiting the potential of this resource (15, 16). By increasing awareness, engagement, and utilization of primary healthcare services among this group, it can enhance individual health outcomes, strengthen population health, and contribute to the sustainability and efficiency of the healthcare system (13).

Face-to-face interventions are effective in improving health outcomes among older adults, especially those who are vulnerable and isolated. These interventions allow active reach to home-dwelling older adults who may not engage with healthcare resources, enabling early detection of unmet needs and timely support (17). Home-based interventions directly assess the health and quality of life of participants, as well as the availability of household resources and social support. This context enables more personalized, practical, and effective care planning, ensuring that interventions are tailored to individuals’ real-life needs and circumstances (18).

The initial point of engagement in home visits is followed with digital health communication. There is high smartphone penetration among older adults in Hong Kong, with 90.7% of individuals aged 65 and above owning a smartphone (19). Hence, digital outreach can provide a practical, affordable, and efficient approach to engage the large aging population without the constraints of mobility and time. Digital educational outreach is often used in health communication to induce health behavior changes, it has been used to increase healthcare service uptake (20), improve healthcare appointment attendance (21, 22), promote screening (23), and promote health promotion activities (24, 25). Most of these digital healthcare communications primarily depend on text messaging, and their effectiveness is mixed (20–22). With comparable cost and resources, multimedia integration strategies that combine video, photo, and text can improve user engagement, enhance visual appeal, promote sharing with family and friends, and boost intervention effectiveness (26, 27). In which, a healthcare study on 2,509 patients educating about colorectal cancer screening reported a significant doubling in screening rates when using videos and text compared to text alone (27), demonstrating benefits of multimedia approaches.

Older adults tend to have reduced digital literacy and possess lower education levels. A total of 53.1% of individuals aged 65 or over have a primary education level or below (28). Our previous study also showed an average eHEALS score of 21.41 out of 40, representing low digital literacy among older adults (15, 16). To generate highly tailored multimedia content for overcoming barriers faced by older adults in accessing health information, artificial intelligence will be utilized. Artificial intelligence is beneficial for personalizing health communication content based on target demographics (29, 30), it can also simplify and clarify complex health information for improved understanding (31). The evidence in Generative AI for support of digital behavioral change interventions development is inconsistent (32, 33), though older participants have reported to be more responsive and persuaded by the tailored AI-generated content (29). Whether AI-generated personalized digital intervention content can increase District Health Center membership uptake is unknown.

Both face-to-face and digital interventions are based on the Information-Motivation-Behavioral Skills (IMB) model conceptual framework, a widely recognized, behavior-focused model commonly applied in health education initiatives (34). The IMB framework includes four key elements, which are knowledge, motivation, behavioral skills, and behaviors. It proposes that these components work both independently and together to shape health-related actions. This model was selected because it offers a structured and holistic approach for addressing the multifaceted challenges that older adults face in increasing utilization of primary healthcare (35).

This pilot study will deliver an intergenerational education outreach, with home-based intervention and AI-generated digital health communication, to promote enrolment to local District Health Centers. This will translate into greater success in the adoption and continuity of primary healthcare service utilization behaviors, such as health promotion activities, preventive screenings, and regular check-ups, ultimately overall improved wellbeing. The specific objectives of this study are to: (1) develop a digital intervention message bank personalized for older adults for DHC uptake using artificial intelligence; and (2) evaluate the feasibility, acceptability, and preliminary effectiveness of the education outreach intervention for increasing the proportion of District Health Center members among older adults in Hong Kong.

## Methods

### Study design

**Figure 1:**
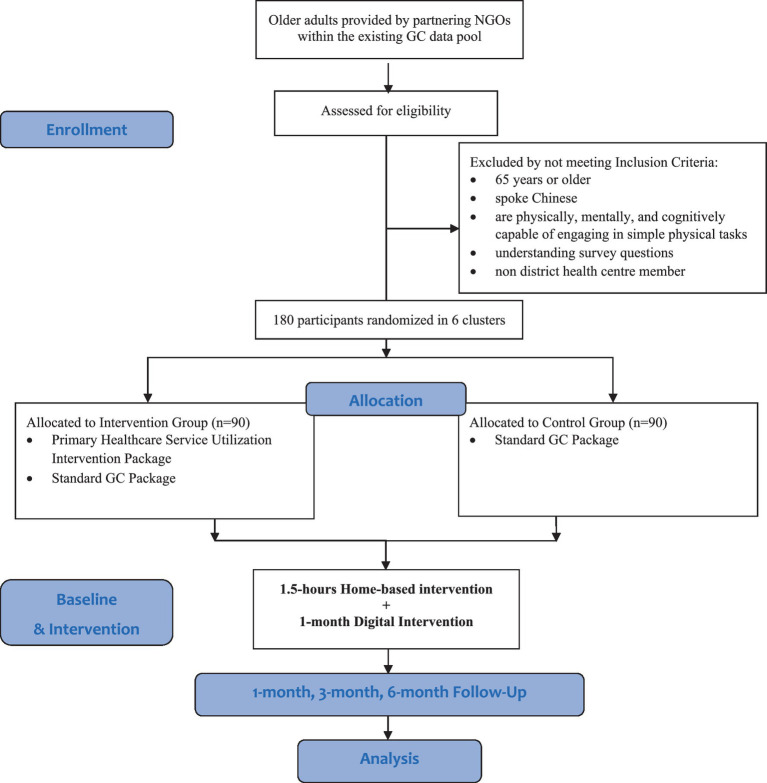
CONSORT (consolidated standards of reporting trials) flow diagram of the study.

The study draws on the data pool collected through the *Generations Connect* (GC) Project, a large-scale community-based initiative aiming to train over 1,000 full-time healthcare students to provide community service and health behavioral coaching to 10,000 older adults in Hong Kong (15, 16). This study is a pilot, two-arm, parallel cluster randomized controlled trial study to evaluate the feasibility, acceptability, and exploratory effectiveness of a hybrid intergenerational home and digital intervention for increasing the proportion of District Health Center membership among older adults in Hong Kong. Data collection will be both offline (face-to-face for baseline) and online (via telephone for 1-month, 3-month, 6-month follow-up) in Nov 2025 - Jul 2026. The study design adheres to the Consolidated Standards of Reporting Trials (CONSORT) reporting guideline (see Figure 1).

### Participants

Older adults will be recruited through collaborations with local non-government organizations (NGOs) covering 18 districts across Hong Kong. Older adults are eligible if they (1) are 65 years or older, (2) can speak and read Chinese (including Putonghua and Cantonese), (3) are physically, mentally, and cognitively capable of engaging in simple physical tasks, (4) can understand survey questions, (5) and not a District Health Center member. Recruitment will be conducted in partnership with community centers, senior clubs, and healthcare facilities, which will provide lists of interested individuals.

### Randomization, allocation, and blinding

The recruiting NGO health centers in three geographical areas will be randomized, using a computer-generated sequence, to either the intervention or control arm in a 1:1 ratio. Due to the nature of the study design and intervention, blinding of participants and intervention providers is not feasible. However, the outcome assessors, statistical analysts, and other investigators involved remain blinded to group allocation.

### Student interventionists training

Student interventionists are current students enrolled full-time at the University of Hong Kong (HKU) and fluent in Cantonese. Students will be recruited through internal university emails and on-campus promotional materials, such as posters. Recruited students will undergo training provided by research and clinical faculty members before conducting the home-based intervention. A specially designed curriculum will be developed to build knowledge and competence among facilitators on three key domains: (1) knowledge of the research protocol and background, (2) technique in delivering the hybrid intergenerational home and digital intervention on primary health care utilization, and (3) application of the general intervention package (15, 16) (see Table 1).

**Table 1:** The curriculum content for training student interventionists.

Theme	Training content
Research protocol	The ageing population and the new primary healthcare journey in Hong Kong Effective communication techniques with older adults Home-based intervention protocols and general safety practice guidelines The effectiveness of AI and digital technology in health interventions for older adults
Primary healthcare service utilization	Overview of primary healthcare resources in Hong Kong
General intervention package *Non-communicable disease prevention, mental health and wellbeing, digital health literacy, infectious disease prevention*	Knowledge on non-communicable disease prevention through the promotion of healthy lifestyles Strategies for building positive mental health and wellbeing Assistance with digital devices and mobile-based eHealth services Infectious disease behavioral preventive measures

#### Development of the AI-generated digital intervention based on the IMB model

The 1-month digital intervention will be generated, aiming to promote awareness and utilization of primary healthcare services, particularly those offered by DHCs. Twelve of the health-related messages will be sent through the instant messaging platform WhatsApp 3 times per week. This aims to reinforce older adults’ knowledge of DHCs gained during the in-person intervention and provide further motivation for utilizing the available services.

The digital intervention is structured into four sequential weekly themes, with each week targeting a key component of the District Health Center primary healthcare model. The first is primary prevention, which introduces the health promotion activities provided. The second is secondary prevention, and it explains the importance of regular health assessment. The third is tertiary prevention, this promotes chronic disease management and rehabilitation support. The final week provides an overall summary, and inserts information on DHC enrolment requirements (see Table 2).

**Table 2:** Framework of intergenerational digital intervention on primary healthcare service utilization.

Day	Component	Digital intervention content
Week 1—primary prevention		
Mon	Information	Introduction to healthy lifestyle promotion activities
Wed	Motivation	Reflection on suitable health promotion services and healthy routines
Fri	Behavioral skills	Guide for access to health promotion services
Week 2—secondary prevention		
Mon	Information	Explanation on health assessments of hypertension and diabetes screening
Wed	Motivation	Evaluation on personal health status
Fri	Behavioral skills	Guide for access to health assessments
Week 3—tertiary prevention		
Mon	Information	Information on chronic disease management and rehabilitation services
Wed	Motivation	Reflection on priorities related to disease risk
Fri	Behavioral skills	Guide for access to chronic disease management and rehabilitation interventions
Week 4—summary		
Mon	Information	Overview of DHC appointment process
Wed	Motivation	Prompt encouraging enrolment and scheduling of DHC appointments
Fri	Behavioral skills	Final reminder with enrolment details and contact information for DHC services

Each week will include three messages aligned with the IMB Model, delivered on Monday, Wednesday, and Friday, with each message targeting a key component of the IMB Model. The Information component will use videos to deliver core health information through accessible and engaging media. The Motivation component will be delivered through infographics and will inspire and motivate behavior change through behavior change theory-based content. The Behavioral Skills component will consist of text messages providing practical support to facilitate action, such as access guidelines, center addresses, and telephone numbers.

To ensure ease of use, videos and infographics would be delivered as files that can be viewed directly within the WhatsApp interface without requiring external links. During home visits, participants will receive guided WhatsApp setup and comprehensive hands-on demonstrations for the viewing of all message types. Ongoing support is provided throughout the intervention period through WhatsApp group and accessible helplines.

Generative Pre-trained Transformer 4 (GPT-4) will be employed as the primary platform for the generation of a large 60-message intervention bank. The format would include text messages, infographic content, and video frameworks. The AI model will be guided through role-specific prompting as a healthcare researcher, with detailed task descriptions, content specifications, target audience information, and output rules. Official District Health Centers documentations would be provided for the AI model as reference.

The content of text messages and the framework of infographics and videos will be generated by GPT-4, followed by design and fine-tuning for maximum effectiveness (29). Project research assistants will check and adjust the materials for factual accuracy, contextual relevance, and overall readability. Given that the target audience consists of older adults with limited comprehension levels, the content will be kept simple through the limitation of words and minimization of technical terminologies. All refined AI-generated materials will undergo review by qualified public health professionals to ensure compliance with established health communication standards.

**Figure 2:**
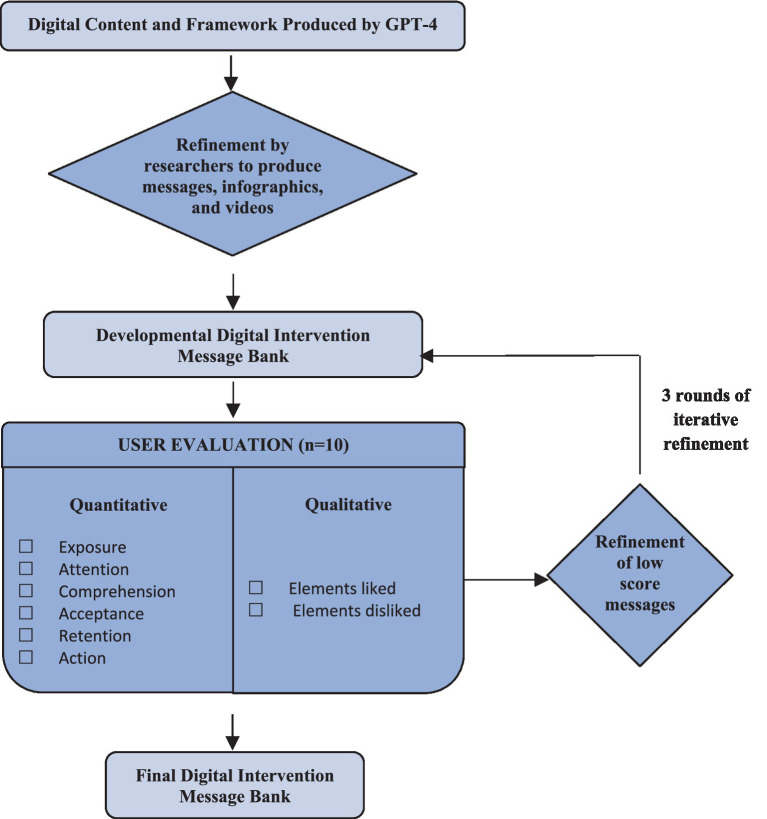
The AI-generated and user-centered digital intervention design process. The initial digital intervention message bank undergoes three iterative rounds of refinement, incorporating both quantitative and qualitative user feedback from older adults, to develop the final, optimized message bank.

Subsequently, a user-centered design process will be conducted (see Figure 2). There will be three iterative rounds of message evaluation, involving 10 participants per round. Each round will include both quantitative and qualitative assessments to guide message refinement. For the quantitative assessment, participants will independently rate each message using a 5-point Likert scale (1 = strongly disagree to 5 = strongly agree) across six dimensions based on McGuire’s Model of Communication and Persuasion: exposure, attention, comprehension, acceptance, retention, and action (36) (see Table 3). For the qualitative assessment, participants will engage in WhatsApp-based feedback, where they will be prompted to provide brief comments on each message. They will be asked to identify specific elements they liked, as well as to elaborate on any negative experiences if applicable.

**Table 3:** Older adults quantitative and qualitative evaluation on AI-generated messages.

Item	Contents					
Exposure	The material is shown clearly on my mobile device.	1 = Strongly disagree	2 = Disagree	3 = Neutral	4 = Agree	5 = Strongly agree
Attention	The material looks good and catches my attention.	1	2	3	4	5
Comprehension	The material is easy to understand.	1	2	3	4	5
Acceptance	The material is clear and non-judgmental.	1	2	3	4	5
Retention	The material is memorable and stays with me.	1	2	3	4	5
Action	The material motivates me to use DHC services.	1	2	3	4	5

Iterative refinement will be conducted using a review–refine–retest cycle. After each round, messages will be revised based on both quantitative ratings and qualitative input and then retested in the subsequent round. First, all messages with low mean scores on any evaluation dimension will be identified for major revision. Then, to guide refinement, qualitative feedback will be analyzed to identify recurring issues and themes. These comments will be categorized into message-specific and general remarks. The message-specific comments will guide targeted major revisions to individual messages, while the broad comments will be applied as minor revisions across all messages of the same format.

### Control group: general intervention

The control group will receive standard care, which is adapted from the intervention package as outlined in our recent publications, referred to as the *Standard Generations Connect Package* (15, 16). The Standard GC Package is delivered through two major approaches: a home-based intervention and a digital intervention. The home-based intervention is a 90-min face-to-face session, during which trained student interns collect health assessment data and deliver theme-based, tailored health interventions. The interventions are focused on four key areas: non-communicable disease (NCD) prevention, psychosocial health, digital health literacy, and infectious disease prevention.

- Non-communicable disease prevention: Reduce the risk of non-communicable diseases by encouraging a healthy lifestyle. Older adults will receive guidance on physical activity, healthy dietary choices, smoking cessation, and alcohol moderation.
- Mental health and wellbeing: Enhance the mental wellbeing of older adults by promoting positive thinking, emotional resilience, and social engagement. Participants will be encouraged to adopt relaxation techniques to manage stress. They will also be introduced to practical strategies and resources that support psychosocial health and wellbeing.
- Digital Health literacy: Strengthen older adults’ ability to find, evaluate, and use electronic health information by guiding them to use WhatsApp for contacting health professionals and connecting with family or friends, using QR code scanners to acquire health information from printed materials, and downloading and using mobile health service applications, such as HA Go and Electronic Health Record Sharing System (eHealth).
- Infectious disease prevention: Enhance knowledge and practices related to seasonal influenza by educating on hygiene, handwashing techniques, government vaccination benefits, and mask-wearing.

Participants will receive the *standard Generations Connect Blue Folder*, which contains relevant health education leaflets. Student interventionists will explain the leaflet content in a simplified and personalized manner, suited to the comprehension levels of older adults. To prepare them for the upcoming digital intervention, a dedicated WhatsApp group will be created for each participant. Students will guide participants on how to access and use WhatsApp and ensure they are able to navigate the app confidently. Over the following month, participants in the control group will receive daily digital messages via WhatsApp, providing general health advice based on the four themes of the *Standard Generations Connect Package*.

### Intervention group: primary healthcare service utilization package

The intervention group will receive the *Primary Healthcare Service Utilization Intervention Package,* in addition to the *Standard Generations Connect Package*. The main focus for the intervention group is primary healthcare service engagement; it will promote the use of primary healthcare services, particularly those offered by District Health Centers, to support health promotion, regular health assessments, chronic disease management, and community rehabilitation. Older adults will receive guidance on the benefits of utilizing these services and will be encouraged to undergo routine health check-ups. Participants will strengthen their understanding of DHCs, increase their awareness of available services, and be motivated to actively utilize them.

The intervention is similarly delivered through two major approaches: home-based intervention and a digital intervention. The home-based intervention is a 90-min face-to-face session, during which trained student interns collect health assessment data and deliver theme-based, tailored health interventions. The digital intervention is a 1-month follow-up support involving daily messages via instant messaging apps (e.g., WhatsApp) that include health-related infographics, videos, and personalized advice.

#### Home-based intervention

The face-to-face proactive home-based intergenerational intervention lasts up to 90 min. Trained student interventionists will conduct baseline health assessments and deliver interventions in pairs. Written consent will be obtained from participants before the assessment begins. The assessment process is flexible, adapting to older adults’ responses. They will conduct the baseline assessment in a semi-structured manner, engaging participants in natural conversations while covering questionnaire items. The questions are read aloud to older adults, and responses are provided verbally.

Participants in the intervention group will receive a *PHC Green Folder*, in addition to the standard *GC Blue Folder*. The *PHC Green Folder* contains targeted materials related to primary healthcare services, including health education leaflets with QR codes linking to online resources, aimed at promoting awareness and encouraging the use of DHC services. Student interventionists will explain the leaflet content and tailor discussions to address the specific health needs of each older adult. The home-based intervention materials are aligned with the Information-Motivation-Behavioral Skills Model (see Table 4). At the end of the intervention, student interventionists will set up individual one-on-one WhatsApp chat groups with the older adults to facilitate the subsequent one-month digital intervention.

**Table 4:** Framework of home-based intervention on primary health care service utilization.

Component	Intervention details
Information	*DHC Information Pamphlet*—Covers DHC services, the importance of primary healthcare, and the benefits of regular health assessments and health promotion.
Motivation	*Experience Sharing Pamphlet*—Presents experiences of older adults who have positively benefited from using DHC services, based on qualitative findings from the *Generations Connect* project.
Behavioral skills	*Visit Guide Pamphlet*—Provides maps, transportation options, and opening hours to help participants locate their district-specific DHC, along with guidance on securing a booking.
Behavior	*Health Decision-making Pamphlet*—Encourages older adults to make informed health decisions and take actions to improve their wellbeing, strengthening self-efficacy.

## Outcomes

### Feasibility outcomes

Feasibility and acceptability indicators will be measured at three-month follow-up (37). Feasibility outcomes will be assessed using recruitment rate, retention rate, dose received through percentage of message views and participant engagement through interaction with digital messages. Other indicators include dose delivered, in which intervention fidelity is measured using a protocol checklist and visit time record. Acceptability outcomes include participant satisfaction with the home-based intervention and the 4-week follow-up digital intervention.

### Primary outcomes

The primary outcome is the proportion of older adults who become DHC members at 3 months. It is a single-item self-report measure of whether participant has joined the District Health Center (Yes/No).

### Secondary outcomes

Secondary outcomes assessed are the proportion of older adults utilizing DHC services, experience and satisfaction with DHC services, health status, lifestyle risk factors, eHealth literacy, and infectious disease prevention behaviors after 3 months. A single-item measure will determine the DHC service utilized by older adults. The item will examine which services are accessed across four core DHC areas of health promotion, health assessments, chronic disease management, and community rehabilitation. Emphasis is placed on the utilization of health promotion activities and regular health assessments, as the utilization of these services is independent of participants’ health status. Health status consists of physical health, quality of life, overall psychological health, loneliness, depression, and anxiety. Modifiable lifestyle risk factors for non-communicable chronic diseases include physical activity, diet, alcohol consumption, and smoking habits.

### Data collection

The study will use a mixed-methods design to construct a comprehensive understanding of the educational health communication intervention on DHC enrollment.

#### Quantitative measurements

Participants’ data will be collected using a self-administered survey at baseline and a telephone survey at 1, 3, and 6 months after the intervention. The baseline questionnaire includes demographics (9 items) and a self-constructed scale on DHC membership and utilization (17 items). Other scales on habits and wellbeing are also included in the questionnaire, such as healthcare service utilization (2 items), physical health (7 items), exercise habits [7 items, using validated IPAQ-SF (38)], dietary practices (5 items), smoking [3 items, using validated HSI (39)], alcohol consumption [3 items, using validated AUDIT-C (40)], psychosocial health and lifestyle [28 items, using validated WHO-5 (41), UCLA 3-item loneliness scale (42), PHQ-9 (43), and GAD-7 (44)], e-health literacy and mobile phone/app usage [29 items, using validated DHLI (45)], and infectious disease prevention behavior [15 items, using IPBS-I (46)].

The 1-month, 3-month, and 6-month follow-up surveys included similar items (demographics excluded), the 3-month follow-up would assess feasibility and acceptability. The 1-month, 3-month, and 6-month follow-up survey will be conducted via telephone.

### Qualitative study

To gain a deeper understanding of participants’ perspectives, a descriptive qualitative study with semi-structured interviews would be conducted. They would be individual interviews or couple-based focus group discussions. These qualitative methods aim to explore older adults’ attitudes, perceptions, and the barriers and facilitators they face in utilizing primary healthcare services. The interviews will also gather insights into their views on the impact of the intervention on their health outcomes and behaviors. Feedback will be collected regarding the strengths and limitations of the intervention, which includes home-based and digital interventions.

### Statistical analyses

#### Sample size planning

No formal sample size justification was conducted since this pilot feasibility trial is primarily intended to investigate feasibility parameters and exploratory effectiveness, in order to inform the sample size for a later full-scale trial. Feasibility trials are generally underpowered, and formal calculations are typically not recommended (47). Based on considerations of variability in sociodemographic representation, two NGO partners would be recruited from each of the three geographical regions in Hong Kong, including Hong Kong Island, Kowloon, and the New Territories. A sample size of 30 is expected per cluster. With six clusters, this would result in a total of 180 participants. Given the limited power and cluster, any observed outcomes on intervention effectiveness would be interpreted with caution.

For the qualitative study, a subsample of 18, representing approximately 10% of participants, will be selected. Three people from each cluster will be purposively selected for qualitative data collection. Maximum variation purposive sampling techniques will be used to select participants based on their demographic information, including gender, income, and education level. This sample size would be sufficient to gather a diverse range of experiences and perceptions to identify key thematic patterns.

#### Data analysis

SPSS Statistics (version 25.0) will be used for data analysis, and an intention-to-treat analysis will be performed. Descriptive statistics will be used to describe demographic variables. For continuous variables, data will be reported as mean ± standard deviation (SD) if normally distributed, or median with interquartile range (IQR) for non-normal distributions. Categorical variables will be presented as frequencies and percentages. To examine exploratory effectiveness given the small numbers of clusters, the binary primary outcome will be evaluated with cluster-level analysis and generalized linear mixed models (48). Missing data will be addressed using multiple imputation with 20 imputations. Statistical significance will be set at a two-tailed *p*-value of 0.05. Statistical analyses will account for the potential effects of intracluster correlation.

For the qualitative interview data, all audio-recorded interviews will first be transcribed verbatim in Cantonese. Two researchers will then independently conduct thematic analysis, with any discrepancies discussed and resolved by a third senior researcher when necessary. NVivo (version 14) will be used for data analysis.

## Discussion

### Limitations

Key limitations are in the DHC membership verification, smartphone-dependent intervention delivery, and generalizability of findings. The primary outcome of DHC membership is assessed through participant self-report since direct access to official enrolment records is not available to the research team for objective verification. The questionnaire will include questions regarding participation in activities within the DHC to help assess enrolment status, as membership is required for participation.

Intervention delivery depends on smartphone ownership and WhatsApp use. The intervention is only available to individuals who have access to a smartphone and are able to use WhatsApp. Individuals with very limited digital literacy may experience barriers to viewing and engaging with the digital intervention. This exclusion of non-smartphone users can also disproportionately affect highly socioeconomically disadvantaged individuals who lack mobile phone ownership or internet access, and those who do not use smartphones by choice. This raises ethical considerations regarding the exclusion of populations who are already socially and digitally marginalized. Such exclusion may compromise equitable access to the intervention and should be carefully considered for the planning of future implementation.

The generalizability among older adults is limited due to the unique target participant sample of underprivileged older adults recruited through NGOs, which serve as one of their main community healthcare sources. This limits the representativeness of the sample for the broader well-off older adult population using other healthcare resources. In addition, Hong Kong has unique healthcare system and digital features that limit generalizability. The tertiary sector of healthcare is well developed, alongside an emerging policy focus on primary health care system and District Health Center network. In terms of smartphone penetration, it is notably high (90.7%) among local older adults. As a result, transferability of study results beyond Hong Kong should be cautiously considered, accounting for differences in healthcare development and digital access conditions.

### Implications

This pilot study has important implications in future research and public health strategies for changes in health-seeking behaviors amongst ageing populations. By combining face-to-face outreach with AI-generated digital interventions, it brings a promising methodology for overcoming barriers and resistance among older adults for primary healthcare service enrolment.

A more personalized approach for integrating artificial intelligence into health communication targeting populations with low digital and health literacy will be demonstrated. The use of a quantitative and qualitative user-centered refinement process addresses a critical gap in AI-generated digital interventions. This digital intervention generation protocol is anticipated to improve responsiveness to the specific needs of vulnerable populations and bring higher effectiveness on behavioral health outcomes.

Current study relies on a university-community partnership. If the study results show feasibility within the academic sector, the digital intervention generation and refinement framework and the interventionist training protocol could be adapted and extended to local healthcare centers. More trained personnel in the community would be equipped to deliver the intervention for DHC uptake. This improves sustainability and scalability beyond academic settings, supporting a shift toward a prevention-focused healthcare system in Hong Kong.

## Conclusion

This pilot cluster randomized controlled trial aims to evaluate the feasibility, acceptability, and effectiveness of a hybrid intergenerational home and AI-generated digital intervention for District Health Center enrolment. The study will explore the implications of these findings for health policy and research, emphasizing the potential of digital intervention created by artificial intelligence to address the unique needs of aging populations and promote their behavior change for increasing primary health service uptake.

## References

[1] World Health Organization World Health Organization Global Strategy and Action Plan on Ageing and Health (2017). Geneva: World Health Organization.

[2] Census and Statistics Department Hong Kong Population Projections for 2022–2046 (2023). Hong Kong: Census and Statistics Department, The Government of the Hong Kong Special Administrative Region.

[3] Department of Health EHS - Coping with Chronic Illness (2023). Hong Kong: Department of Health, The Government of the Hong Kong Special Administrative Region.

[4] Health Bureau Primary Healthcare Blueprint (2022). Hong Kong: Health Bureau, The Government of the Hong Kong Special Administrative Region.

[5] Mak IL Liu KSN Wong ZCT Xu VYH Yu EYT Ha TKH . Evaluation of the effectiveness and cost-effectiveness of the chronic disease co-care (CDCC) pilot scheme: a study protocol. BMC Prim Care. (2025) 26:73. doi: 10.1186/s12875-025-02765-6, 40108515 PMC11921508

[6] Health Bureau Hong Kong’s Domestic Health Accounts (DHA) 2022/23 (2025). Available online at: https://www.healthbureau.gov.hk/statistics/en/dha.htm (Accessed July 12, 2026)

[7] Ho MK. Strengthening primary care in Hong Kong: fostering continuity of care from a health system perspective. Hong Kong Med J. (2020) 26:543–5. doi: 10.12809/hkmj198368, 33350972

[8] Ng TKC Fong BYF Yee HHL. Ageing with Dignity in Hong Kong and Asia, Holistic and Humanistic Care 2022 (2022). Singapore: Springer Singapore.

[9] Xiong X Li VJ Huang B Huo Z. Equality and social determinants of spatial accessibility, availability, and affordability to primary health care in Hong Kong, a descriptive study from the perspective of spatial analysis. BMC Health Serv Res. (2022) 22:1364. doi: 10.1186/s12913-022-08760-2, 36397059 PMC9670047

[10] District Health Centre Healthcare Service Providers (2019). Available online at: https://www.dhc.gov.hk/en/healthcare_service_providers.html#scope-of-service (Accessed July 12, 2026)

[11] Lin J Bates S Allen LN Wright M Mao L Chomik R . Uptake of patient enrolment in primary care and associated factors: a systematic review and meta-analysis. BMC Prim Care. (2025) 26:76. doi: 10.1186/s12875-025-02779-0, 40119278 PMC11927268

[12] Bates SM Lin J Allen L Wright M Kidd M. The impact of patient enrolment in primary care on continuity and quality of care around the world, 2014-2024, and lessons for Australia: a scoping review. Med J Aust. (2025) 222:462–71. doi: 10.5694/mja2.52648, 40231620 PMC12088321

[13] Han TC Lin HS Chen CM. Association between chronic disease self-management, health status, and quality of life in older Taiwanese adults with chronic illnesses. Healthcare (Basel). (2022) 10:609. doi: 10.3390/healthcare10040609, 35455788 PMC9027156

[14] Zhang M Zhao M Wei X. Research progress on community health management model for older adults with chronic diseases and multiple morbidities. Br J Hosp Med (Lond). (2024) 84:1–9. doi: 10.12968/hmed.2024.0256, 39475018

[15] He AWJ Yuan R Luk TT Wang KMP Chan SSC. Boosting digital health engagement among older adults in Hong Kong: pilot pre-post study of the generations connect project. JMIR Form Res. (2025) 9:e69611. doi: 10.2196/69611, 40340793 PMC12080964

[16] He AWJ Yuan R Chan SSC. Socio-demographic and behavioral predictors of multiple-dose COVID-19 vaccine uptake among older adults in Hong Kong: a community-based cross-sectional study of the generations connect project. Vaccine. (2025) 61:127308. doi: 10.1016/j.vaccine.2025.127308 40460652

[17] Duan Y Yang M Wang Y Cheng S Liang W Lippke S . Effects of a blended face-to-face and eHealth lifestyle intervention on physical activity, diet, and health outcomes in Hong Kong community-dwelling older adults: a randomized controlled trial. BMC Public Health. (2025) 25:2145. doi: 10.1186/s12889-025-23311-0, 40495124 PMC12150582

[18] Wong FK Chow SK Chan TM Tam SK. Comparison of effects between home visits with telephone calls and telephone calls only for transitional discharge support: a randomised controlled trial. Age Ageing. (2014) 43:91–7. doi: 10.1093/ageing/aft123, 23978408 PMC3861338

[19] Census and Statistics Department Thematic Household Survey Report No. 77 (2023). Hong Kong: Census and Statistics Department, The Government of the Hong Kong Special Administrative Region.

[20] Levine DM Kakani P Mehrotra A. Randomized controlled study using text messages to help connect new medicaid beneficiaries to primary care. NPJ Digit Med. (2021) 4:26. doi: 10.1038/s41746-021-00389-5, 33589706 PMC7884833

[21] Robotham D Satkunanathan S Reynolds J Stahl D Wykes T. Using digital notifications to improve attendance in clinic: systematic review and meta-analysis. BMJ Open. (2016) 6:e012116. doi: 10.1136/bmjopen-2016-012116, 27798006 PMC5093388

[22] Percac-Lima S Singer DE Cronin PR Chang Y Zai AH. Can text messages improve attendance to primary care appointments in underserved populations? J Health Care Poor Underserved. (2016) 27:1709–25. doi: 10.1353/hpu.2016.0157, 27818433

[23] Raine R Moss SM von Wagner C Atkin W Hans IK Howe R . A national cluster-randomised controlled trial to examine the effect of enhanced reminders on the socioeconomic gradient in uptake in bowel cancer screening. Br J Cancer. (2016) 115:1479–86. doi: 10.1038/bjc.2016.365, 27875518 PMC5155361

[24] Runsen Z Yueying X Tieguang H Guoan Y Yuan Z Li C . Short message service usage may improve the public's self-health management: a community-based randomized controlled study. Health Sci Rep. (2022) 5:e850. doi: 10.1002/hsr2.850, 36189410 PMC9498217

[25] Kwan RYC Salihu D Lee PH Tse M Cheung DSK Roopsawang I . The effect of e-health interventions promoting physical activity in older people: a systematic review and meta-analysis. Eur Rev Aging Phys Act. (2020) 17:7. doi: 10.1186/s11556-020-00239-5, 32336996 PMC7175509

[26] Neuhauser L Kreps GL. EHealth communication and behavior change: promise and performance. Soc Semiot. (2010) 20:9–27. doi: 10.1080/10350330903438386

[27] Coronado GD Ruiz E Torres-Ozadali E Thompson JH Rivelli JS Thibault A . Video text messaging is needed to deliver patient education about preventive care in the United States. PLOS Digit Health. (2023) 2:e0000258. doi: 10.1371/journal.pdig.0000258, 37253020 PMC10228759

[28] Census and Statistics Department Hong Kong 2021 Population Census - Thematic Report: Older Persons (2023). Hong Kong: Census and Statistics Department, The Government of the Hong Kong Special Administrative Region.

[29] Chu H Liu S. Generating targeted and tailored health communication narratives with AI. Risk Anal. (2025) 45:3505–18. doi: 10.1111/risa.70076, 40755067

[30] Zhu Y Long Y Wang H Lee KP Zhang L Wang SJ. Digital behavior change intervention designs for habit formation: systematic review. J Med Internet Res. (2024) 26:e54375. doi: 10.2196/54375, 38787601 PMC11161714

[31] Willis VC Thomas Craig KJ Jabbarpour Y Scheufele EL Arriaga YE Ajinkya M . Digital health interventions to enhance prevention in primary care: scoping review. JMIR Med Inform. (2022) 10:e33518. doi: 10.2196/33518, 35060909 PMC8817213

[32] Hussain SA Schmälzle R Lim S Bouali N. Comparing AI and human-generated health messages in an Arabic cultural context. Glob Health Action. (2025) 18:2464360. doi: 10.1080/16549716.2025.2464360, 39963959 PMC11837920

[33] Xia D Song M Zhu T. A comparison of the persuasiveness of human and ChatGPT generated pro-vaccine messages for HPV. Front Public Health. (2024) 12:1515871. doi: 10.3389/fpubh.2024.1515871, 39886390 PMC11780328

[34] Fisher WA Fisher JD Harman J. "The information-motivation-Behavioral skills model: a general social psychological approach to understanding and promoting health behavior". In: Social Psychological Foundations of Health and Illness (2003). p. 82–106. Malden, MA: Blackwell Publishing Ltd. doi: 10.1002/9780470753552.ch4

[35] Chang SJ Choi S Kim S-A Song M. Intervention strategies based on information-motivation-behavioral skills model for health behavior change: a systematic review. Asian Nurs Res. (2014) 8:172–81. doi: 10.1016/j.anr.2014.08.002

[36] Rice R Atkin C McGuire W. (2013). Mcguire’s classic input–output framework for constructing persuasive messages. In Mcguire’s classic input–output framework for constructing persuasive messages (Fourth Edition ed., pp. 133-145). SAGE Publications, Inc., doi: 10.4135/9781544308449.n9

[37] Saunders RP Evans MH Joshi P. Developing a process-evaluation plan for assessing health promotion program implementation: a how-to guide. Health Promot Pract. (2005) 6:134–47. doi: 10.1177/1524839904273387, 15855283

[38] Lee PH Macfarlane DJ Lam TH Stewart SM. Validity of the international physical activity questionnaire short form (IPAQ-SF): a systematic review. Int J Behav Nutr Phys Act. (2011) 8:115. doi: 10.1186/1479-5868-8-115, 22018588 PMC3214824

[39] Diaz FJ Jané M Saltó E Pardell H Salleras L Pinet C . A brief measure of high nicotine dependence for busy clinicians and large epidemiological surveys. Aust N Z J Psychiatry. (2005) 39:161–8. doi: 10.1080/j.1440-1614.2005.01538.x, 15701065

[40] Bush K Kivlahan DR McDonell MB Fihn SD Bradley KA. The AUDIT alcohol consumption questions (AUDIT-C): an effective brief screening test for problem drinking. Ambulatory care quality improvement project (ACQUIP). Alcohol use disorders identification test. Arch Intern Med. (1998) 158:1789–95. doi: 10.1001/archinte.158.16.1789, 9738608

[41] Fung SF Kong CYW Liu YM Huang Q Xiong Z Jiang Z . Validity and psychometric evaluation of the Chinese version of the 5-item WHO well-being index. Front Public Health. (2022) 10:872436. doi: 10.3389/fpubh.2022.872436, 35433612 PMC9005828

[42] Russell DW. UCLA loneliness scale (version 3): reliability, validity, and factor structure. J Pers Assess. (1996) 66:20–40. doi: 10.1207/s15327752jpa6601_2, 8576833

[43] Kroenke K Spitzer RL Williams JB. The PHQ-9: validity of a brief depression severity measure. J Gen Intern Med. (2001) 16:606–13. doi: 10.1046/j.1525-1497.2001.016009606.x, 11556941 PMC1495268

[44] Spitzer RL Kroenke K Williams JB Löwe B. A brief measure for assessing generalized anxiety disorder: the GAD-7. Arch Intern Med. (2006) 166:1092–7. doi: 10.1001/archinte.166.10.1092, 16717171

[45] Xie L Hu H Lin J Mo PKH. Psychometric validation of the Chinese digital health literacy instrument among Chinese older adults who have internet use experience. Int J Older People Nursing. (2024) 19:e12568. doi: 10.1111/opn.12568, 37831059

[46] Heo ML Jang YM. Development and validation of the infection prevention behavior scale of individuals (IPBS-I) for the general population. J Multidiscip Healthc. (2021) 14:2791–802. doi: 10.2147/JMDH.S334154, 34675528 PMC8502068

[47] Thabane L Ma J Chu R Cheng J Ismaila A Rios LP . A tutorial on pilot studies: the what, why and how. BMC Med Res Methodol. (2010) 10:1. doi: 10.1186/1471-2288-10-1, 20053272 PMC2824145

[48] Thompson JA Leyrat C Fielding KL Hayes RJ. Cluster randomised trials with a binary outcome and a small number of clusters: comparison of individual and cluster level analysis method. BMC Med Res Methodol. (2022) 22:222. doi: 10.1186/s12874-022-01699-2, 35962318 PMC9375419

